# DEFiNE: A Method for Enhancement and Quantification of Fluorescently Labeled Axons

**DOI:** 10.3389/fnana.2018.00117

**Published:** 2019-01-11

**Authors:** Jeanne M. Powell, Nicholas W. Plummer, Erica L. Scappini, Charles J. Tucker, Patricia Jensen

**Affiliations:** ^1^Neurobiology Laboratory, National Institute of Environmental Health Sciences, National Institutes of Health, United States Department of Health and Human Services, Durham, NC, United States; ^2^Signal Transduction Laboratory, National Institute of Environmental Health Sciences, National Institutes of Health, United States Department of Health and Human Services, Durham, NC, United States

**Keywords:** axon density, axonal projections, connectivity, Code: ImageJmacro, autofluorescence, digital enhancement, lipofuscin, fluorescent artifact

## Abstract

Visualization and quantification of fluorescently labeled axonal fibers are widely employed in studies of neuronal connectivity in the brain. However, accurate analysis of axon density is often confounded by autofluorescence and other fluorescent artifacts. By the time these problems are detected in labeled tissue sections, significant time and resources have been invested, and the tissue may not be easy to replace. In response to these difficulties, we have developed Digital Enhancement of Fibers with Noise Elimination (DEFiNE), a method for eliminating fluorescent artifacts from digital images based on their morphology and fluorescence spectrum, thus permitting enhanced visualization and quantification of axonal fibers. Application of this method is facilitated by a DEFiNE macro, written using ImageJ Macro Language (IJM), which includes an automated and customizable procedure for image processing and a semi-automated quantification method that accounts for any remaining local variation in background intensity. The DEFiNE macro is open-source and used with the widely available FIJI software for maximum accessibility.

## Introduction

Detailed knowledge of neural connectivity is fundamental to our evolving understanding of how the brain processes information. Our ability to tease apart the brain’s complexity has been dramatically enhanced by the advent of viral and genetic methods for labeling small populations of neurons and mapping their axonal projections (Bang et al., 2012; Robertson et al., 2013, 2016; Beier et al., 2015; Plummer et al., 2015, 2017; Schwarz et al., 2015; Niederkofler et al., 2016; Uematsu et al., 2017; Poulin et al., 2018). However, these methods have also increased the challenges of analysis. Successful quantification of fluorescently labeled axons depends on a high signal-to-noise ratio, with bright axons and relatively low background. When labeling is restricted to sparse projections from a few neurons, autofluorescence and other background artifacts can constitute the bulk of the fluorescent signal, significantly impeding manual axon tracing and rendering automated quantification techniques wholly unreliable. Furthermore, tissue is often a limited reagent when axon quantification is part of a larger analysis of experimental animals, restricting researchers’ ability to optimize labeling conditions. Thus, a procedure is required for obtaining reliable quantification from fluorescent images, even when fibers are sparsely distributed and in the presence of autofluorescent artifacts.

Autofluorescence is influenced by many factors, including the age of the animal and fixation techniques, and it usually has broad excitation and emission spectra, making it highly disruptive of fluorescence microscopy (Van de Lest et al., 1995; Billinton and Knight, 2001). A variety of experimental approaches for dealing with autofluorescence and other fluorescent artifacts have been developed, but all have limitations. Dyes for blocking autofluorescence (e.g., Sudan Black B) are suitable for imaging of neuronal cell bodies but may quench desired fluorescence in fine axonal fibers (Romijn et al., 1999; Schnell et al., 1999). Photo-irradiation of sections has been used with some success to bleach autofluorescence before immunolabeling (Viegas et al., 2007; Duong and Han, 2013; Kumar et al., 2015; Sun and Chakrabartty, 2016); however, free-floating sections are difficult to manage, and long treatment times are required for thick sections. Furthermore, photo-irradiation cannot be used to bleach fluorescent artifacts resulting from immunolabeling (aggregated secondary antibodies, non-specific binding, etc.) due to the likelihood of weakening the intended signal. Similarly, chemical treatments used to reduce autofluorescence resulting from aldehyde-based fixation (Clancy and Cauller, 1998) will have no effect on immunolabeling artifacts.

As an alternative to these experimental manipulations, digital processing may be used after tissue is imaged. Several methods for digitally enhancing, tracing, or quantifying axons have recently been described (Grider et al., 2006; Dehmelt et al., 2011; Kim et al., 2015; Paletzki and Gerfen, 2015; Haas et al., 2016; Patel et al., 2018), but their ability to deal with a wide variety of fluorescent artifacts is limited. One simple method for digitally removing background fluorescence, including autofluorescence, from digital images is to apply a threshold cutoff, removing all pixels with intensity below the threshold value. The threshold can be determined by sampling the image in regions lacking labeled axons and/or by visual inspection of the image before and after application of different threshold values (Mossberg et al., 1990). However, this procedure is of limited value if background fluorescence is uneven, and it breaks down completely when intensity of autofluorescent structures approaches or exceeds that of axonal fibers. Application of a median filter or Gaussian blur to the image can reduce uneven background and enhance signal (Paletzki and Gerfen, 2015; Noller et al., 2016), but these techniques risk obscuring fine, low intensity axons and will have little effect on bright, highly structured autofluorescence like lipofuscin granules. Fluorescence spectrum can also be used to identify and remove artifacts such as objects with broad emission spectrum that appear in multiple channels during confocal microscopy (Oh et al., 2014). Alternatively, the broad excitation spectra of autofluorescent molecules can be exploited by collecting digital images after excitation at two different wavelengths, one that excites both the specific fluorescent label and autofluorescent molecules, and a second that excites only autofluorescence (Van de Lest et al., 1995; Belichenko et al., 1996). The autofluorescence-only image is then digitally subtracted from the first image. Unfortunately, this method is impractical when multiple fluorophores are used, because no autofluorescence-specific excitation wavelength is available.

Here, we describe Digital Enhancement of Fibers with Noise Elimination (DEFiNE), a method for autofluorescence detection and removal from digital images that is compatible with experiments using multiple fluorophores. A macro to facilitate application of this method uses the open-source image analysis software package FIJI (Schindelin et al., 2012). Instead of exploiting the broad excitation range of autofluorescent molecules, DEFiNE takes advantage of their broad emission range, similar to a procedure previously used in fluorescence activated cell sorting (FACS; Roederer and Murphy, 1986). This autofluorescence subtraction is combined with removal of background fluorescence based on morphology, and semi-automated quantification of axonal fibers in the processed images, providing a versatile digital assistant for axon mapping projects.

## Materials and Methods

### Sample Preparation

To test DEFiNE on images containing multiple fluorophores, we used tissue from mice heterozygous for recombinase-responsive fluorescent indicator alleles and recombinase driver alleles which restrict expression of enhanced green fluorescent protein (EGFP) and/or tdTomato to subpopulations of noradrenergic neurons, including their axonal projections (Plummer et al., 2015). All animal work was performed in accordance with the recommendations in the Guide for the Care and Use of Laboratory animals of the National Institutes of Health. The protocols were approved by the Animal Care and Use Committee (ACUC) of the National Institute of Environmental Health Sciences. Mice were anesthetized with sodium pentobarbital and transcardially perfused with 4% paraformaldehyde (PFA) in ice-cold 0.1 M phosphate buffered saline (PBS). After dissection, brains were post-fixed by immersion overnight in 4% PFA in PBS at 4°C, rinsed in PBS, and equilibrated in 30% sucrose in PBS for 48 h at 4°C. For immersion fixation, fresh brains were immersed in ice cold 4% PFA in PBS overnight, followed by equilibration in 30% sucrose in PBS. The cryoprotected brains were embedded in Tissue Freezing Medium (General Data Company, Cincinnati, OH, USA) and sectioned on a Leica CM3050 S cryostat (Leica Biosystems, Buffalo Grove, IL, USA). 40-μm-thick free-floating sections were stored in 30% sucrose/30% ethylene glycol in PBS at −80°C.

For immunofluorescent labeling, free-floating sections were washed with 0.1% Triton X100 in PBS (PBST), followed by incubation in 0.1 M glycine in PBST (30 min, room temperature) and 5% normal goat serum in PBST (1 h, room temperature). The sections were labeled with chicken anti-GFP (1:10,000; ab13970, Abcam, Cambridge, MA, USA), rabbit anti-dsRed (1:1,000; 632496, Clontech Laboratories, Mountain View, CA, USA), and mouse anti-norepinephrine transporter (NET; 1:1,000; 1447-NET, Phosphosolutions, Aurora, CO, USA) primary antibodies, followed by goat anti-chicken Alexa Fluor 488 (1:1,000; A11039, Thermo Fisher Scientific, Waltham, MA, USA), goat anti-rabbit Alexa Fluor 568 (1:1,000; A11036, Thermo Fisher), and goat anti-mouse Alexa Fluor 633 (1:1,000; A21052, Thermo Fisher) secondary antibodies. After washing, the sections were stained with Neurotrace 435/455 blue fluorescent Nissl stain (1:50, N21479, Thermo Fisher) and mounted onto Superfrost Plus microscope slides (Thermo Fisher). Cover slips were applied using Prolong Diamond antifade mountant (Thermo Fisher).

### Image Collection

**Figure 1:**
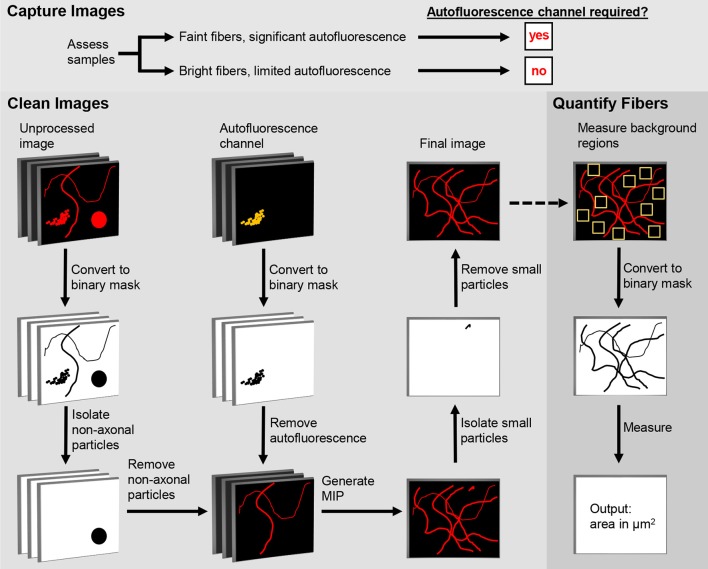
Digital Enhancement of Fibers with Noise Elimination (DEFiNE) workflow. Autofluorescence removal by the Clean Images function depends on capture of an autofluorescence channel during imaging. When samples have brightly labeled axons and limited noise, the autofluorescence channel can be omitted during imaging and processing. The Clean Images and Quantify Fibers functions may be used separately or in tandem, as indicated by the dashed arrow.

The first step in the DEFiNE process (Figure 1) is to qualitatively assess the samples to determine full imaging parameters. Samples with brightly labeled axonal fibers and faint autofluorescence require no special imaging setup. For samples with low signal-to-noise ratios (e.g., sparse fibers and bright, dense autofluorescence) the imaging setup should include an autofluorescence channel that is captured while scanning the tissue at the excitation wavelength of one of the fluorescent labels. Images are collected at the expected emission range of the label fluorophore and simultaneously at a longer wavelength range in which autofluorescence is observed but the label fluorophore does not emit. In multi-fluorophore experiments, the second emission range (the autofluorescence channel) can overlap one of the other fluorescent labels on the tissue, because that fluorophore is not excited.

In our experiments, we imaged four fluorescent labels and tested three excitation/emission combinations for the autofluorescence channel. For far-red Alexa Fluor 633, we excited with a 633 nm helium-neon laser (pinhole setting 1 airy unit) and collected the emission signal with a 640–758 nm filter. For red Alexa Fluor 568, we excited with a 561 nm diode-pumped solid-state laser and collected with a 571–633 nm filter, and also with a 640–758 nm filter for the autofluorescence channel. For green Alexa Fluor 488, we excited with a 488 nm argon-krypton laser and collected with a 491–562 nm filter, and also with a 571–633 nm filter for autofluorescence channel. For the blue fluorescent Nissl stain, we excited with a 405 nm diode laser and collected with a 415–470 nm filter, and also with a 491–562 nm filter for the autofluorescence channel. Z-stack images through the full thickness of each section were collected using a Zeiss LSM 880 inverted confocal microscope (Carl Zeiss Microscopy, Thornwood, NY, USA) with Plan-Apochromat 20×/0.8 M27 objective (2.06 μs pixel dwell time, 0.830 × 0.830 × 2 μm voxel size, and line averaging set to 4) or Plan-Apochromat 40×/1.3 Oil M27 objective (1.03 μs pixel dwell, 0.208 × 0.208 × 0.800 μm voxel, and line averaging 4).

### Preparing Images for DEFiNE

The DEFiNE macro consists of two functions, Clean Images and Quantify Fibers (Figure 1). Images to be processed can be in any format supported by FIJI’s Bio-Formats Importer (Linkert et al., 2010). Stacks should be cropped to remove z-slices imaged above or below the tissue, because empty z-slices may interfere with thresholding by DEFiNE’s Clean Images function. This cropping will also exclude autofluorescent particles such as dust or secondary antibody conglomerates that lie between the tissue and coverslip. Tile scan images of large brain regions should be stitched, preferably with the software used to collect the images, before DEFiNE processing. For testing DEFiNE’s Quantify Fibers function, we used output from the Clean Images function. Axons can also be quantified in images that have not been processed through the Clean Images function, but each input image must be a single-channel maximum intensity projection (MIP).

### DEFiNE Image Processing

The Clean Images function of DEFiNE removes background autofluorescence and other fluorescent artifacts from multi-channel images of fluorescently labeled axons. The function consists of three automated steps: large particle removal, autofluorescence removal, and small particle removal. The first two steps are performed on a z-stack, and the last is performed after generation of a MIP. In images with low signal-to-noise ratios, as when bright lipofuscin obscures sparse axonal fibers, the best results are obtained using all three steps. However, when axon labeling is very bright and only scattered artifacts are present, users can forgo the autofluorescence removal and use the two steps that remove artifacts based on their morphology.

Default settings were optimized using fluorescently labeled noradrenergic axons, but the Clean Images function also includes a Settings Optimization sub-function which guides users who wish to customize the settings to accommodate different fluorescence intensities or axon morphologies. This sub-function processes a representative image from the user-defined input folder and displays the results of the particle subtraction and autofluorescence subtraction steps of DEFiNE processing (see below). The user can mark inappropriately removed axons and/or incorrectly retained autofluorescence, and in response the macro will print recommended settings. When the user is satisfied with the results, the optimized custom settings are automatically fed into the Clean Images function and all images are processed accordingly.

*Large particle removal* subtracts undesirable fluorescence based on size (μm^2^) and circularity (range 0–1, where 1 represents a perfect circle). The DEFiNE user interface permits users to select up to five combinations of minimum size and circularity for removal. Default size/circularity settings are 40/0.17, 25/0.32, 15/0.40, 10/0.70, and 5/0.80. As circularity decreases, the minimum size of particles to be removed should be increased to avoid removing fibers. Four iterations of the subtractions are performed using thresholds of 1.5, 2, 2.5, and 3 standard deviations above average pixel intensity. For each iteration, images are converted to a binary mask and particles that meet the user-defined minimum size/circularity requirements are identified and removed using the “Analyze Particles” function and Image Calculator “AND” function in FIJI, respectively.

*Autofluorescence removal* subtracts autofluorescent structures captured in the autofluorescence channel of the multi-channel image. Each z-slice of the autofluorescence channel is converted to a binary image, and the Image Calculator “AND” function in FIJI is used to digitally subtract the autofluorescent pixels from the remaining channels. The default threshold cutoff for the binary image is 1 standard deviation above average pixel intensity. At this threshold, intensely labeled fibers that fluoresce faintly in the autofluorescence channel may be removed during processing. Therefore, the DEFiNE interface permits the user to select a higher threshold as needed to minimize loss of bright fibers while maximizing removal of autofluorescence. Alternatively, the autofluorescence removal step can be omitted, and fluorescent artifacts can be subtracted using large and small particle removal exclusively.

*Small particle removal* subtracts fluorescent objects too small to be axons, which result from random noise or incomplete removal of larger fluorescent structures during earlier processing steps. The DEFiNE macro converts the processed z-stack to a MIP using FIJI’s Z-Project function, and then generates a binary mask at a threshold 1 standard deviation above the mean pixel intensity. By default, particles with area ≤1 μm^2^ and circularity 0.99–1 are selected using FIJI’s “Particle Analysis” function and are removed using the Image Calculator “AND” function. The DEFiNE user interface allows both criteria to be changed if necessary.

After processing, each channel is saved as its own image file in a FIJI-generated folder named “DEFiNE_Processed_Images_month-day-year.” Following DEFiNE processing, images used to prepare figures for this article were modified only by adjustment of brightness or contrast across the entire image.

### DEFiNE Fiber Quantification

To quantify axonal fibers in a single-channel MIP image, the DEFiNE Quantify Fibers function allows the user to select ten 12 × 12 μm regions where no labeled axons are visible and calculates the mean pixel intensity and standard deviation of those regions. A threshold is set at 4 standard deviations above mean pixel intensity, and the area of the image (μm^2^) occupied by pixels with intensity above that threshold is recorded. This procedure is performed on every image in the selected folder, and the data is saved in a text file. Additionally, binary images at the calculated threshold are saved in a FIJI-defined subfolder named “DEFiNE_Quantified_Fibers_month-day-year.”

### Code Accessibility

The DEFiNE macro for FIJI is available for download at https://figshare.com/s/1be5a1e77c4d4431769a. DEFiNE has been tested and found to be compatible with FIJI version 1.51w.

### Manual Fiber Tracing and Quantification

To manually trace axonal fibers in unprocessed and DEFiNE processed images, we used the NeuronJ plug-in for FIJI (Meijering et al., 2004). For consistency, all manual tracing was performed by the same investigator. GraphPad Prism 7 (GraphPad Software Inc., La Jolla, CA, USA) was used for statistical analyses (paired and unpaired *t*-tests) and creation of graphs.

## Results

To test the ability of DEFiNE to enhance visualization and quantification of axonal fibers in images containing significant artifacts, we imaged mouse brain sections with noticeable and problematic autofluorescence. Sections from mice expressing EGFP and tdTomato in subpopulations of noradrenergic neurons were first immunolabeled to increase fluorescence intensity in axons, using green Alexa Fluor 488- and red Alexa Fluor 568-conjugated secondary antibodies, respectively. In addition, to increase the range of fluorophores and label intensities in our test images, noradrenergic axons were also immunolabeled with an anti-NET primary antibody and a far-red Alexa Fluor 633-conjugated secondary antibody, and neuron cell bodies were labeled with a blue fluorescent Nissl stain. Next, we selected sections containing autofluorescent lipofuscin, hemosiderin, visible blood vessels, and/or other fluorescent debris for analysis.

**Figure 2:**
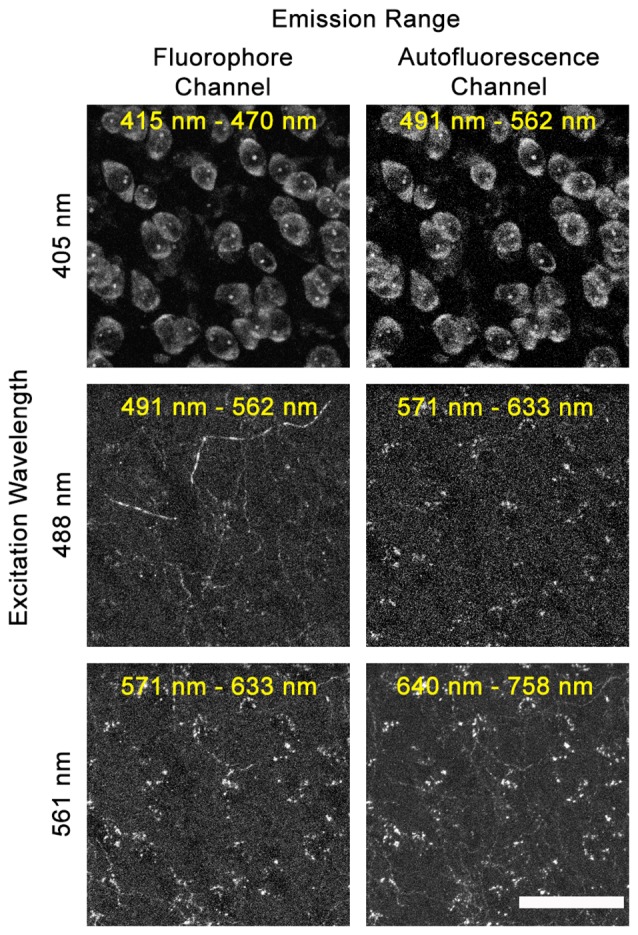
Selecting the optimal autofluorescence channel. Images show fluorescent emission at selected wavelengths after excitation using 405 nm, 488 nm, or 561 nm lasers. In our experiments using blue, green, red, and far-red fluorophores, the optimum autofluorescence channel was created by exciting the tissue with a 488 nm laser and imaging fluorescence between 571 nm and 633 nm. When exciting with the 405 nm laser, the broad emission spectrum of the blue NeuroTrace Nissl overlaps the autofluorescence channel. When exciting with the 561 nm laser, the broad excitation range of far-red Alexa Fluor 633 leads to labeled fibers being captured in the 640–758 nm autofluorescence channel. Scale bar: 50 μm.

Full DEFiNE processing requires that multi-channel digital images include an autofluorescence channel that samples background fluorescence but not labeled axons. As an initial step in designing DEFiNE, we determined the optimal wavelengths for the autofluorescence channel. In our experiments, using blue, green, red, and far-red fluorophores, we found that the best autofluorescence channel was created by exciting the tissue with a 488 nm laser and collecting fluorescent emission in the red range from 571 nm to 633 nm, outside the emission range of green Alexa Fluor 488 (Figure 2). An autofluorescence channel created by exciting with the 405 nm laser and collecting emissions in the green range (Figure 2, top row) was not successful due to the very wide emission spectrum of the blue fluorescent Nissl stain, which resulted in detection of labeled cell bodies in the autofluorescence channel. Exciting with the 561 nm laser and collecting in the far-red range was similarly unsatisfactory, because the wide excitation spectrum of far-red Alexa Fluor 633 resulted in detection of labeled axons in the autofluorescence channel (Figure 2, lower right). In principle, multiple autofluorescence channels might be required to detect and remove all autofluorescent artifacts; however, we found the 488/571–633 nm excitation/emission combination captured most of the autofluorescence observed in images collected using the other lasers (Figure 2, compare center right and lower right). A few axons, very brightly labeled with EGFP/Alexa Fluor 488, were faintly visible in the autofluorescence channel, but appropriate thresholding permitted retention of these axons when autofluorescence was subtracted. Although the 571–633 nm collection range of our autofluorescence channel overlaps the emission range of Alexa Fluor 568, no axons labeled with that fluorophore were captured in the autofluorescence channel, because they were not excited by the 488 nm laser.

**Figure 3:**
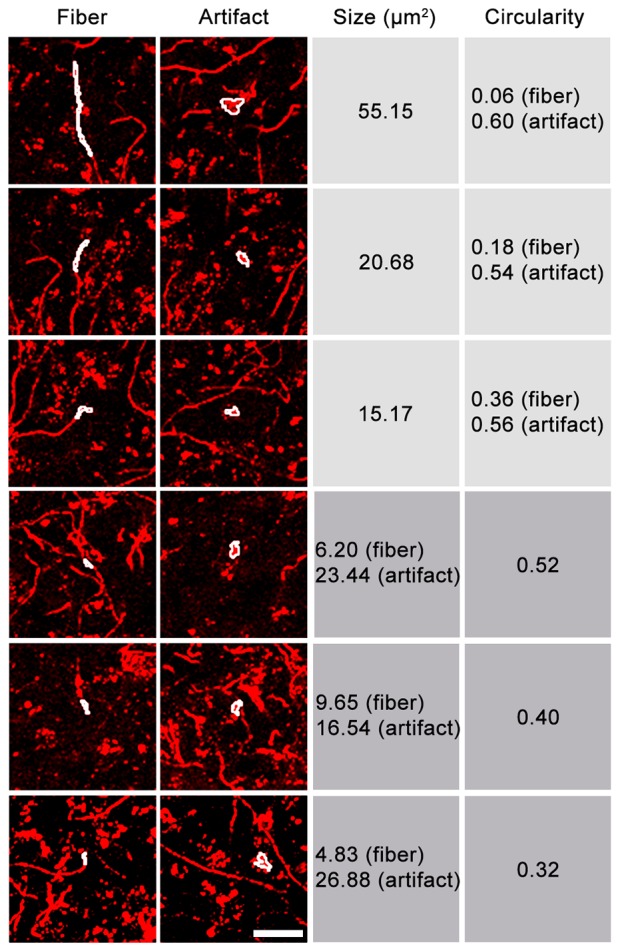
Relationship between size and circularity of axonal fibers and fluorescent artifacts. Representative images contain Alexa Fluor 568 (tdTomato)-labeled axonal fibers and fluorescent artifacts, including autofluorescence. FIJI’s “Analyze Particles” function was used to identify fibers and fluorescent particles of equal size or equal circularity (white outlines). Fluorescent particles tend to exhibit higher circularity than fibers of equal size. Particles and short fiber segments of equal circularity are often different sizes. Considering both characteristics allows a greater proportion of fluorescent noise to be removed. Scale bar: 25 μm.

Because not all fluorescent artifacts will be captured in an autofluorescence channel, we next tested the possibility of removing fluorescent structures from the images based on morphology. While size or circularity alone proved to be insufficient selection criteria, considering both characteristics allowed axons to be distinguished from many fluorescent artifacts (Figure 3). Highly circular structures could be removed, even if their size equaled that of fibers in the image, while more linear structures (e.g., blood vessels) could be removed if their area exceeded that of axons. Fluorescent artifacts whose size/circularity overlapped that of axonal fibers could not be removed using this processing method and remained in the image unless autofluorescence removal was applied. This procedure is incorporated into DEFiNE’s Large Particle Removal step.

**Figure 4:**
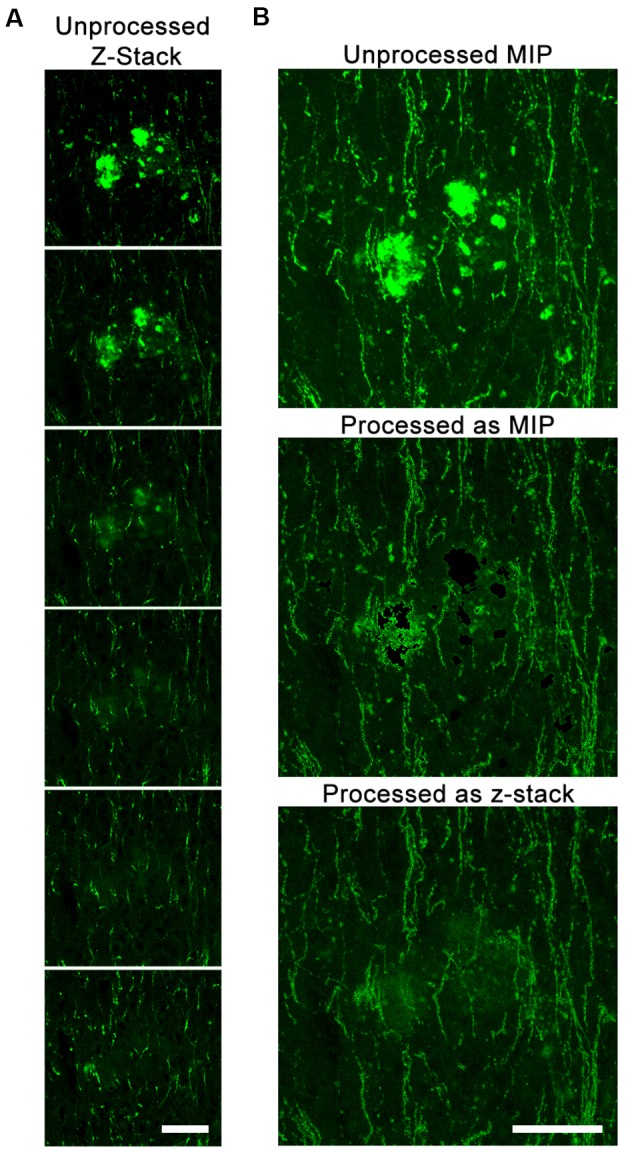
DEFiNE processing of z-stack images allows for maximal preservation of axonal fibers.** (A)** Individual z-slices within one z-stack image from the vertical limb of the diagonal band of Broca. Autofluorescent debris is present in the first three z-slices, and Alexa Fluor 488 enhanced green fluorescent protein (EGFP)-labeled axonal fibers are visible at the same location in the final three. **(B)** Maximum intensity projections (MIPs) of the unprocessed image, the image processed as a MIP, and the image processed as a z-stack. Fibers lost when processing a MIP are preserved when processing the z-stack. Scale bars: 50 μm.

We also tested whether DEFiNE processing was better applied to z-stack images or MIPs. Because axonal fibers have a very narrow diameter relative to a 40-μm-thick section, we observed fibers in some z-stack images running behind fluorescent artifacts that did not span the entire thickness of the section (Figure 4A). DEFiNE processing of MIPs resulted in a complete loss of signal at the x-y coordinates of the autofluorescence, while processing z-stacks allowed preservation of fibers above or below the autofluorescence and generated a final image with more even background (Figure 4B).

**Figure 5:**
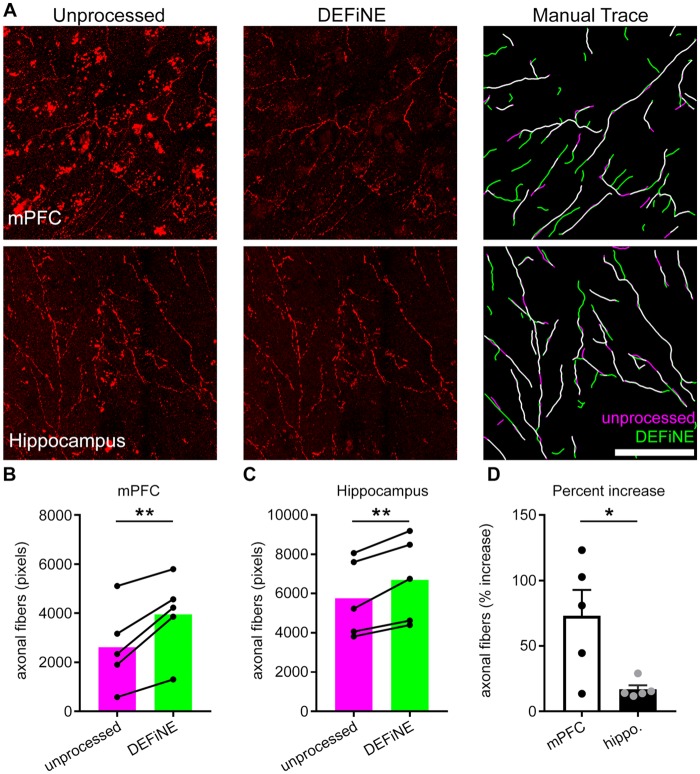
DEFiNE processing does not result in loss of axonal fibers. **(A)** Axonal fibers were manually traced in unprocessed (left) and DEFiNE processed (center) fluorescent images from medial prefrontal cortex (mPFC) and area CA1 of the hippocampus. At right, the fibers traced from the unprocessed image (magenta) are shown with those traced from the processed image (green). Fibers traced in both images appear white. Scale bar: 50 μm. **(B)** Axonal fibers traced before and after DEFiNE processing in five mPFC images are represented as total pixel count (**p = 0.008, paired *t*-test). **(C)** Fibers traced before and after DEFiNE processing in five hippocampus images (**p = 0.007, paired *t*-test). **(D)** Percent increase in fibers observed in DEFiNE processed images relative to unprocessed images. The increase in fiber visibility is greater in cortex (*p = 0.023, unpaired *t*-test).

Next, we investigated whether or not the full DEFiNE process resulted in loss of labeled axons. We manually traced fluorescently labeled noradrenergic fibers in images of medial prefrontal cortex (*n* = 5 mice) and hippocampus (*n* = 5 mice) before and after processing and quantified the results (Figure 5). We observed no loss of fibers. In fact, we detected more fibers after DEFiNE processing in both cortex (*p* = 0.008, paired *t*-test) and hippocampus (*p* = 0.007, paired *t*-test), suggesting that DEFiNE uncovered fibers obscured by fluorescent artifacts in the unprocessed images. The percent increase was higher for the cortex, compared to hippocampus (*p* = 0.023, unpaired *t*-test), likely because the greater density of lipofuscin in the cortex made it more difficult to identify faint fibers in unprocessed images.

**Figure 6:**
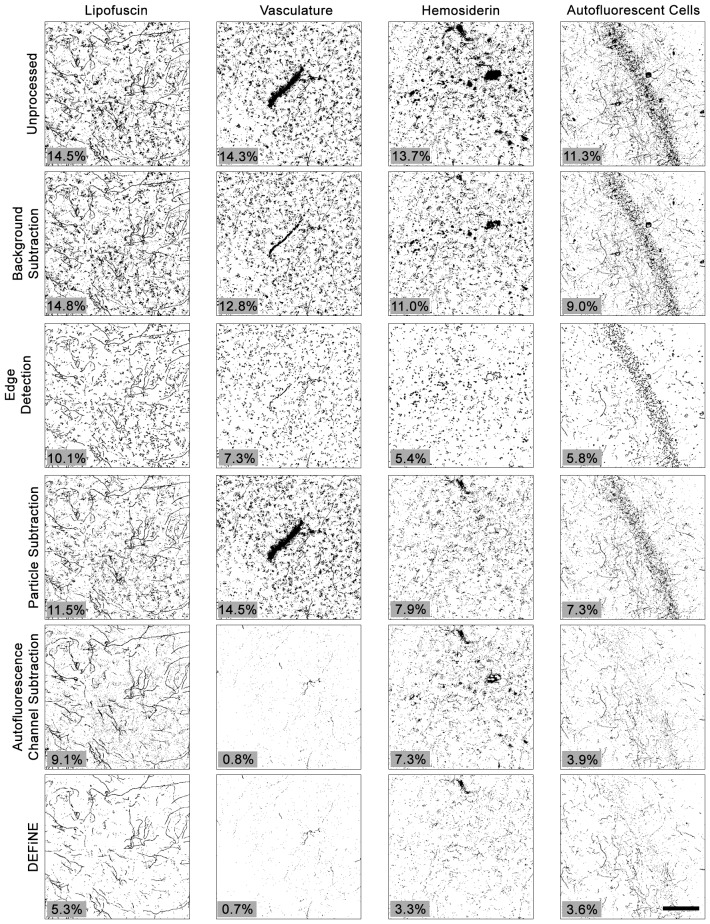
The full DEFiNE process eliminates more fluorescent artifacts than alternate methods for removing background or selecting axonal fibers. Binary images generated by DEFiNE’s Quantify Fibers function show axons and fluorescent artifacts in unprocessed images and after rolling-ball background subtraction (FIJI), Hessian-based edge detection (Grider et al., 2006), morphology-based particle subtraction, autofluorescence channel subtraction, or DEFiNE. The fluorescent signal remaining after processing (black pixels) is indicated as a percentage of the total image area (lower left of each panel). Scale bar: 100 μm.

To further test the value of our new method, we also compared DEFiNE with two alternative digital methods for noise reduction and/or isolation of axonal fibers: the built-in background subtraction function in FIJI and a Hessian-based edge detection method (Grider et al., 2006), using the FeatureJ plug-in for FIJI (Erik Meijering^1^). We tested the ability of background subtraction, Hessian-based edge detection, the individual processing steps from DEFiNE (large particle removal and autofluorescence removal), and the full DEFiNE procedure to isolate axonal fibers in images containing lipofuscin, autofluorescent cells, vasculature, or hemosiderin and fluorescent debris at a stereotaxic injection site (Figure 6). After application of a threshold cutoff, images processed using FIJI’s background subtraction function retained considerable background which constituted the bulk of the signal above threshold (Figure 6, row 2). Edge detection is effective for selecting linear axonal fibers in images with relatively unstructured background noise, but in our images with bright fluorescent artifacts possessing sharply defined edges, it detected the artifacts as efficiently as the axons (Figure 6, row 3). Autofluorescence removal (Figure 6, row 5) eliminated more background than large particle removal (Figure 6, row 4), but the full DEFiNE procedure most effectively reduced the signal attributable to all of the different autofluorescent sources (Figure 6, row 6).

**Figure 7:**
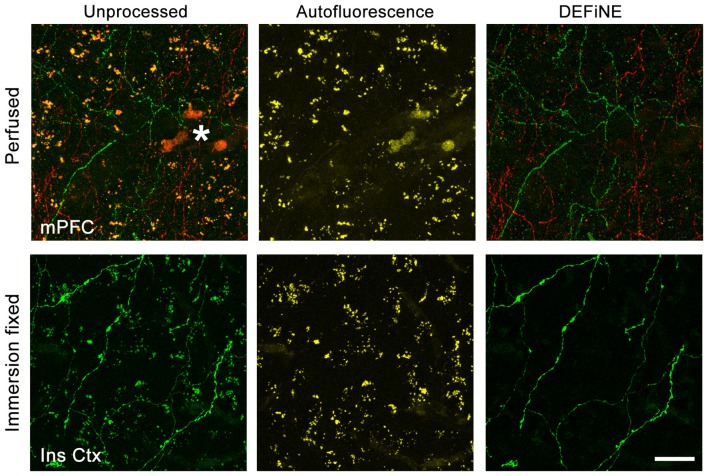
Efficacy of DEFiNE processing does not depend on tissue processing or labeling methods. Results of DEFiNE processing are shown for images collected from tissue fixed by perfusion (top; medial prefrontal cortex, mPFC) or immersion in paraformaldehyde (PFA) solution (bottom; insular cortex, Ins Ctx). The image from perfusion fixed tissue (top) shows noradrenergic axons labeled with Alexa Fluor 488 and Alexa Fluor 568. The image from immersion fixed tissue shows native EGFP fluorescence in noradrenergic neurons expressing the *RC::FLTG* indicator allele (Plummer et al., 2015). Left, unprocessed images in which axonal fibers are obscured by lipofuscin or autofluorescent red blood cells (*). Center, autofluorescence channels. Right, axonal fibers after DEFiNE processing. Scale bar: 20 μm.

Success of fluorescent imaging, particularly of axonal fibers, depends to a large extent on consistent tissue processing and labeling. To confirm the broad applicability of DEFiNE under different processing and labeling protocols, particularly when fixation may have been less than optimal, we imaged brain tissue fixed either by perfusion or by simple immersion in PFA solution. We processed high magnification (40×) images of axonal fibers labeled with native EGFP fluorescence or Alexa Fluor dyes. The fixation protocol and labeling method had no discernible effect on the ability of DEFiNE to remove fluorescent artifacts (Figure 7).

## Discussion

Recent technological advances, including widespread adoption of viral constructs for delivery of genetic tools to label and manipulate the function of targeted neurons (Atasoy et al., 2008; Fenno et al., 2014; Schwarz et al., 2015; Tervo et al., 2016), have increased the need for fluorescent visualization and quantification of axonal fibers. Ideally, fluorescently labeled axons are visualized in tissue from young mice, but the necessary waiting period to allow for expression of a viral payload, together with sometimes prolonged *in vivo* analyses of the injected animals, results in processing and imaging of older tissue with naturally higher levels of autofluorescence (Terman and Brunk, 1998). Fluorescent artifacts are heterogeneous in their origins, properties, and morphologies, so a successful method for cleaning up fluorescent images of axonal fibers must be flexible. DEFiNE’s strength lies in utilizing multiple approaches that complement each other: large particle removal to eliminate fluorescent artifacts—whether autofluorescent or not—that are morphologically distinct from fibers, autofluorescence channel subtraction to remove autofluorescent structures whose size/circularity are indistinguishable to that of axonal fibers, and small particle removal to eliminate random noise. No single step in DEFiNE is sufficient to remove all types of fluorescent artifacts, but together they reliably generate an image in which axonal fibers can be quantified.

Although the optimum autofluorescence channel in our experiments was the 488/571–633 nm excitation/emission combination, the best choice may vary depending on the particular fluorophores in an experiment. Since some axons intensely labeled with Alexa Fluor 488 did fluoresce faintly in our autofluorescence channel, it may be best, when possible, to excite autofluorescence using a wavelength that does not excite any of the fluorophores in the experiment. When this is not possible in multi-fluorophore experiments, the ability to select different threshold cutoffs for autofluorescence removal in the Clean Images function will help minimize loss of axon intensity.

When quantifying fibers in different brain regions, variability in background fluorescence due to inherent differences in brain structure can lead to inconsistent measurements, even after DEFiNE processing, if a single threshold cutoff is used for all images. Similarly, in comparisons among different experimental animals, variation in fluorescence intensity may arise from subtle differences in fixation, tissue processing, and immunolabeling conditions. By guiding the selection of image-specific threshold cutoffs, the Quantify Fibers function accounts for this variability and facilitates axon quantification in large, complex sets of images.

The DEFiNE macro was designed as a user-friendly way to utilize an autofluorescence-only channel and to take advantage of capabilities built into the freely available FIJI software package, in the novel context of processing fluorescent axon images. Our testing indicates that DEFiNE can significantly reduce the intensity of a wide variety of fluorescent and autofluorescent artifacts, while leaving fluorescently labeled axonal fibers intact. Designed to operate in batch-mode, DEFiNE allows users to rapidly process many images, with processed output images and associated data saved into intuitively named and organized folders, and the integrated help function clearly explains each processing step and all opportunities for customizing settings. All of these features are contained in a single open-source user interface which will aid in mapping and quantifying the axonal projections of fluorescently labeled neurons.

## Data Availability

The raw data supporting the conclusions of this manuscript will be made available by the authors, without undue reservation, to any qualified researcher.

## Author Contributions

PJ, JP, and CT designed and planned the project. PJ, JP, NP, CT, and ES performed the experiments. JP, ES, and CT wrote the DEFiNE code. JP, NP, and PJ prepared the figures. JP and NP wrote the manuscript with input from coauthors. PJ and CT edited the manuscript.

## Conflict of Interest Statement

The authors declare that the research was conducted in the absence of any commercial or financial relationships that could be construed as a potential conflict of interest.

## Footnotes

^1^https://imagescience.org/meijering/software/featurej/
